# Enzyme-Based Labeling Strategies for Antibody–Drug Conjugates and Antibody Mimetics

**DOI:** 10.3390/antib7010004

**Published:** 2018-01-04

**Authors:** Georg Falck, Kristian M. Müller

**Affiliations:** Cellular and Molecular Biotechnology, Faculty of Technology, Bielefeld University, Universitätsstr. 25, 33615 Bielefeld, Germany; georg.falck@uni-bielefeld.de

**Keywords:** chemo-enzymatic labeling, armed antibody, antibody coupling, antibody conjugation

## Abstract

Strategies for site-specific modification of proteins have increased in number, complexity, and specificity over the last years. Such modifications hold the promise to broaden the use of existing biopharmaceuticals or to tailor novel proteins for therapeutic or diagnostic applications. The recent quest for next-generation antibody–drug conjugates (ADCs) sparked research into techniques with site selectivity. While purely chemical approaches often impede control of dosage or locus of derivatization, naturally occurring enzymes and proteins bear the ability of co- or post-translational protein modifications at particular residues, thus enabling unique coupling reactions or protein fusions. This review provides a general overview and focuses on chemo-enzymatic methods including enzymes such as formylglycine-generating enzyme, sortase, and transglutaminase. Applications for the conjugation of antibodies and antibody mimetics are reported.

## 1. Introduction

### 1.1. Antibody–Drug Conjugates

**Figure 1:**
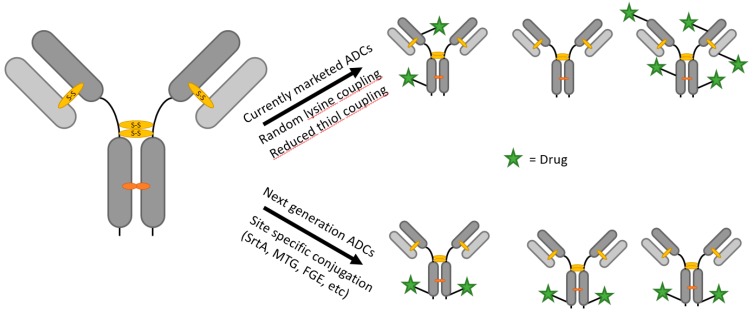
Depiction of unspecific chemical coupling and controlled chemo-enzymatic coupling.

In the fight against cancer, antibody–drug conjugates (ADC) have gained considerable attention, especially since the market release of Kadcyla and Adcetris, which have doubled in yearly sales since 2013, closing in on a billion dollars [1,2]. A vast number of new ADCs are in the pipeline, with seven of them in pivotal clinical trials [3]. A factor fueling the success of ADCs is the promising expansion of the therapeutic window compared to “naked” therapeutic monoclonal antibodies (mABs) and classical chemotherapy. Furthermore, this approach could revive antibodies with suitable affinities yet insufficient cytotoxicity, and highly potent drugs unfavorable for unspecific systemic application. Improvement of therapeutic efficacy has been proven for the trastuzumab–maytansine conjugate T-DM1 on tumor models refractory to trastuzumab [4]. However, said potential therapeutic window is limited by the heterogeneity, stability, and pharmacokinetics of current ADCs and could be restored by site-specific conjugation strategies [5,6]. The drug to antibody ratio (DAR) emerged as a key term in the discussion of the ideal ADC, which has to be high enough to provide sufficient therapeutic potency as well as low enough to not generate heavily drug-modified conjugates with impaired binding properties, stability, and circulation half-life [7,8]. Depending on the properties of the drug, the ideal DAR for most ADCs is between 2 and 4 [7,9]. Coupling strategies for first generation ADCs have been exclusively chemical with random coupling of thiol groups on reduced cysteines, of side chain amine groups on lysines and aldehyde groups on oxidized glycostructures [10]. The abundance of such modification targets leads to a broad distribution of DARs. Subsequent strategies therefore focused on altering interchain disulfide bonds, changing respective cysteines to serines or introducing new free cysteines [11], as utilized for the THIOMAB conjugation strategy [12,13], or modulating the reactivity of specific cysteine residues for the π-clamp strategy [14]. In contrast, this review will focus on enzyme-based strategies to create homogenous antibody–drug conjugates (Figure 1).

### 1.2. Antibody Fragments and Mimetics

Antibody fragments and mimetics were created to generate economical and more convenient high-affinity proteins with alternative properties, though in part similar to those of full antibodies. Intended differences to full antibodies are their smaller size and the possibility to be produced in prokaryotic cells. Some of these mimetics are directly derived from their full antibody counterparts, like antigen-binding fragments (Fab), single-chain variable fragments (scFv) or domain antibodies, such as V_H_H domains (also termed Nanobodies). Nanobodies are derived from camelid heavy–chain–only antibodies and have been used extensively for imaging and even therapeutic applications [15]. They inhibited antigen binding or were loaded with toxins and thereby delayed tumor growth [16,17]. ScFv antibodies for therapy are human or humanized light and heavy chain variable fragments fused by a flexible (glycine-serine) linker. They have shown increased distribution across tumors while retaining antigen affinity. Their potential use for cancer therapy has been discussed [18,19,20] with Blincyto (blinatumomab, anti CD3 scFv–anti CD19 scFv) being an approved drug. Other antibody mimetics consist of conserved protein scaffolds with variable affinity regions mimicking the complementary determining regions of antibodies. Designed ankyrin repeat proteins (DARPins) showed high specificity and good tumor penetration due to their small size and stability [21]. They consist of linker connected turn–helix–helix motifs, typically three plus two capping domains, which form the antigen binding surface. Repebodies, similar to DARPins, are composed of repetitive motifs called leucin-rich repeats organized in a β–strand–turn–α–helix structure [22]. Derived from the α-helical Z domain of staphylococcus protein A, Affibodies, the smallest of the antibody mimetics with around 6 kDa, are mostly used for imaging [23]. A lot of the pros and cons of antibody fragments mimetics are common, such as their ability to penetrate tumors, but also their relatively short half-life in blood circulation and, due to the lack of an Fc part, their missing antibody-dependent cell-mediated cytotoxicity (ADCC) and complement-dependent cytotoxicity (CDC [24,25]. While the shorter circulation time of antibody mimetics narrows the therapeutic window for cancer therapy, it is otherwise beneficial for diagnostic approaches, as the patient’s exposure to substances such as radiolabels is shorter. The enhanced tumor penetration, due to smaller size and typically lower affinities, can be advantageous in the treatment of solid tumors. Whereas whole antibodies usually accumulate between tumor interstitium and tumor surface cells, antibody fragments and mimetics are able to reach the inner tissue faster [24,26]. Antibody mimetics can benefit from modification, like PEGylation and fusion to an albumin-binding domain for longer circulation and site-specific drug coupling for enhanced toxicity.

## 2. Site-Specific Protein Modification Strategies

### 2.1. Formylglycine-Generating Enzymes

**Figure 2:**
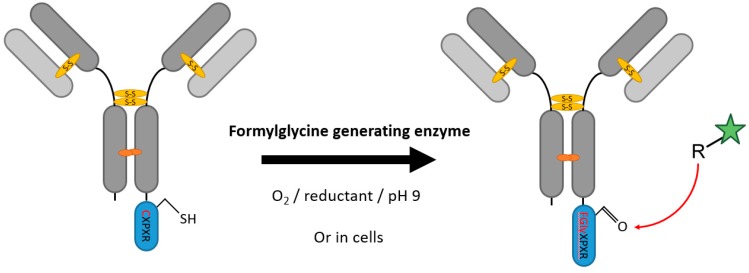
Scheme of an antibody modification with the fromylglycine-generating enzyme. For clarity, the reaction is only depicted in one heavy chain. R represents a moiety with an aldehyde reactive function capable of coupling via, e.g., hydrazino-iso-Pictet-Spengler Ligation, trapped Knoevenagel Ligation, or Wittig reaction.

First found during an investigation of the multiple sulfatase deficiency, formylglycine-generating enzymes (FGE) have the ability to convert cysteines site-specifically to formylglycines, presenting an aldehyde residue unique in proteins and suitable for bio-orthogonal coupling (Figure 2) [27,28]. Their natural substrates are sulfatases bearing a highly conserved hexapeptide, in which the cysteine is converted to formylglycine in the endoplasmic reticulum (ER) [29,30]. FGEs can be found in a variety of organisms, both prokaryotic and eukaryotic, suggesting possible various in vivo as well as in vitro applications. Since its discovery in 2003, human FGE (hFGE) has been predominantly employed in formylglycine conversion in the eukaryotic context [31,32]. When overexpressed, excess hFGE is truncated and secreted from the ER. This secreted form is still catalytically functional and utilizable for in vitro reactions [33]. For prokaryotic systems, different FGEs are available, with *Mycobacterium tuberculosis*, *Streptomyces coelicolor*, and *Thermomonospora curvata* leading the way. Formylglycine, as a novel posttranslational modification, has drawn interest in the field of site-specific modification. Carrico et al. coined the term “aldehyde tag” in 2007 for the short universal FGE recognition motif LCTPSR, later shortened to CXPXR [34,35]. They used this tag for N- or C-terminal protein modification in *E. coli* co-expressed with *M. tuberculosis* FGE [36]. The tag was also introduced in a CHO (Chinese Hamster Ovary) cell system for IgG-Fc and whole antibody modification, showing the preservation of binding functionalities, as well as for labelling of cytosolic and cell surface proteins [37]. For the generation of site-specific coupled antibodies and antibody fragments expression platforms for aldehyde tagged proteins were tested in both E. coli and CHO cells while stably coexpressing hFGE [38]. The effect of positioning of the drug in ADCs has been addressed and aldehyde tag functionality at different sites of an IgG1 antibody has been validated. While aggregation occurred after introduction at certain positions, especially in the CH2 and CH3 domains, for the most part the implemented tags were applicable. The flexibility of the tags use was thus extended to almost all generally accessible areas of the antibody [39]. Formylglycine conversion rates of 75% to over 90% could be achieved, yet total conversion still remains an issue. In an attempt to enhance conversion, different media and copper(II) supplementation were tested yielding higher conversion rates even with 5 µM copper addition. Media composition seems to play a crucial role in conversion results as well, but details remain elusive [40]. Copper supplementation and reconstitution of FGE in vitro have been discussed in the light of new findings regarding the mechanism of the FGE catalysis. Human FGE converts the substrate cysteine after disulfide formation with the Cys-341 in an oxygenase-type reaction requiring molecular oxygen and a reductant [41,42]. Recently copper(I) was proposed as a cofactor [43]. In vitro reconstitution with copper could successfully increase the catalytic efficiency of bacterial FGEs [44,45]. The overall similarity of the mycobacterium tuberculosis and *S. coelicolor* FGE to hFGE including the two cysteines in the catalytic center suggests a common mechanism [46]. Setbacks of the method including precipitation in vitro have been reported, especially in high ionic strength reaction buffers, and diol formation of the formylglycine [44]. At the same time, the possibility of using this enzyme strategy in eukaryotic and prokaryotic environments, as well as both in vitro and in cells, and the number of aldehyde group-based coupling chemistries, e.g., hydrazino–iso–Pictet–Spengler, the Wittig reaction or trapped-Knoevenagel, show its flexibility.

### 2.2. Sortases

**Figure 3:**
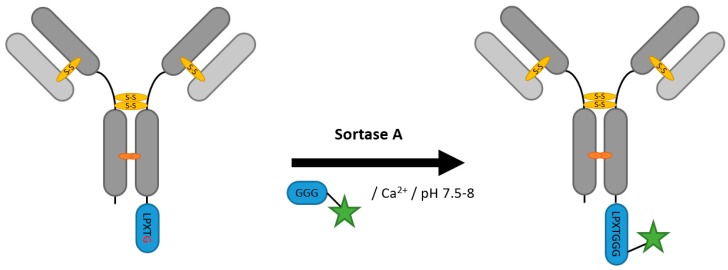
Scheme of an antibody modification with sortase.

Gram positive bacteria produce sortases for the attachment of surface proteins on pentaglycine structures of the peptidoglycane cell wall [47]. *Staphylococcus aureus* sortase A (srtA) is the most frequently used enzyme for site-specific protein labelling with this transpeptidase reaction (Figure 3). Its natural substrate recognition motif is the pentapeptide LPXTG, where X is usually glutamic acid, besides aspartic acid or lysine [48]. In a ping-pong hydrolytic shunt-like mechanism, srtA binds the LPXTG substrate, hydrolyzes the backbone between threonine and glycine, and generates an acyl-enzyme intermediate. The terminal amine of the oligoglycine then acts as a nucleophile, resulting in an amide bond formation with the C-terminal threonine of the substrate [49,50]. The nucleophilic tag can be as short as two or three glycines for the protein ligation to work, making it popular for protein–peptide ligation [51] with biologically as well as chemically synthetized compounds. This has been successfully demonstrated by either using LPXTG or G_n_ at the N- or C-terminus with in vitro modification of purified recombinant proteins and on living cells [52,53,54]. Attempting to circumvent the limitation to either terminus Antos et al. introduced a dual labeling strategy featuring *Streptococcus pyogenes* sortase A. This sortase A is capable of accepting alanines as nucleophiles. The newly ligated C-terminus of the protein will therefore be no substrate for srtA in the second modification step. In combination with srtA twofold modifications via distinct reactions at N- and C-terminus can be conducted [55]. Most of srtA’s use in antibody systems has revolved around diagnostic approaches with single chain antibody fragments derivatized with fluorescent or radioactive markers [56,57,58,59,60,61]. Kornberger and Skerra fused a whole protein, the plant toxin gelonin, to the trastuzumab-Fab with an srtA approach, yielding 50% conversion [62]. Nanobodies have likewise been effectively labeled with fluorophores and cytotoxic payloads [63,64]. Due to the limitation to modifications of either terminus of the target protein, srtA has not been the preferred choice for ADC generation. Considering the so far preferred DAR of four, Beerli et al. could produce Kadcyla- and Adcetris-similar antibodies by separately fusing maytansine- and auristatin-oligoglycine to the light and heavy chain of trastuzumab and brentuximab [65]. They achieved around 80% toxin coupling, corresponding to a DAR of roughly 3.2. Wagner et al. created bispecific antibodies by ligating two full antibodies with a combined strategy of sortase reaction and click chemistry [66]. In the meantime, the repertoire of the method has been extended by diversifying the tag [67] and solving solubility issues and side reactions [56,68]. Furthermore, depsipeptides have been introduced as substrates for N-terminal protein modification to address reversibility of the reaction [69]. Though comparably high enzyme concentrations are needed and the method is limited to in vitro protein modifications, it has been extensively used for successful bio-conjugation.

### 2.3. Transglutaminases

**Figure 4:**
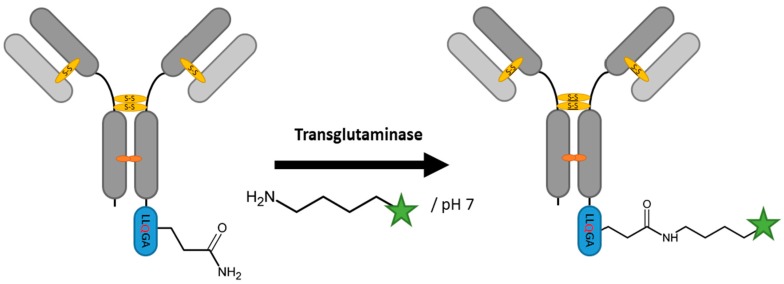
Scheme of an antibody modification with transglutaminase.

Transglutaminases catalyze an acyl-transfer reaction to the side chain of glutamine residues of their protein substrate (Figure 4) [70]. Depending on the acyl donor, this can result in an amide bond between the glutamine and a primary amine, crosslinking between two proteins via a side chain lysine of the donor protein or the deamidation of glutamine [71]. For protein labelling purposes, the acyl-transfer reaction is preferred. While transglutaminases are specific for glutamine on the target protein, the flexibility in terms of the amine containing acyl-donor offers diverse possibilities for modification. In contrast to other protein-ligation strategies, the probe containing reactant is not required to be a peptide and can simply be an alkylamine or an oligoamine as long as it contains a primary amine [72]. Eukaryotic transglutaminases used for protein modification are derived from the guinea pig liver as well as the human transglutaminase 2. However, the bacterial transglutaminase from *Streptomyces mobaraensis* (MTG) is the enzyme of choice due to its independency from calcium and its lower deamidation activity [73]. Transglutaminases display a certain promiscuity when it comes to the glutamine containing recognition sequence, which has spawned a search for a universal minimal tag. Besides phage display derived peptides, different heptapeptide tags with hydrophobic residues N-terminal to the central glutamine have been used [74,75,76]. Farias et al. shortened the tag to an LLQGA motif [77]. A novel strategy was presented by Siegmund et al. by generating a disulfide bridge stabilized handle with an exposed glutamine modelled on a natural MTG substrate reaching ligation efficiencies of 85% [78]. They successfully biotinylated the therapeutic antibody cetuximab, showing the amenability of the tag for possible antibody–drug conjugation. The feasibility of the generation of antibody conjugates with the aid of MTG has been proven repeatedly at different surface accessible sites [6,79,80,81]. The product yields for the latter approaches vary between 80% and 90%. The promiscuity of the enzyme may generate side products, like deamidated glutamine or a transesteferification product, and the need for experimental verification of sites for tag introduction remains. MTG catalysis is mainly used in vitro, but has been done on cells as well [75]. By combining transglutaminase catalysis with engineered cysteine maleimide conjugation, Puthenveetil et al. created a dually labeled antibody reaching a DAR of 4 [82].

### 2.4. Inteins

**Figure 5:**
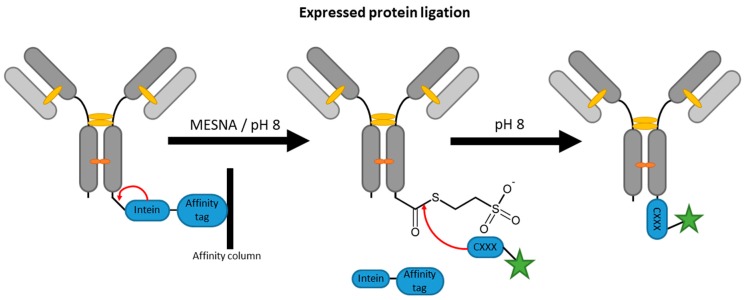
Scheme of an antibody modification by expressed protein ligation. MESNA is the abbreviation for 2-mercaptoethanesulfonate.

**Figure 6:**
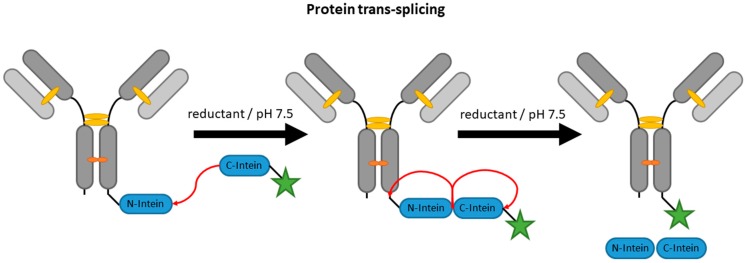
Scheme of an antibody modification by trans-splicing.

Inteins are protein sequences that can cleave themselves off to form a mature protein. Since they bear an internal endoprotease activity, their application is not an enzyme strategy in the classical sense. For site-specific protein modification, inteins are deployed in variations of two different ligation strategies, expressed protein ligation (EPL) (Figure 5) and protein trans-splicing (PTS) (Figure 6). The reaction mechanism involves the formation of a thioester intermediate. It is formed by the N-terminal cysteine of the intein. Subsequently, the transfer of the thioester to an intramolecular cysteine downstream of the intein sequence and the release of the intein, through the generation of a peptide bond between the exteins, completes the reaction. Muir et al. described EPL in 1998, therein exploiting an intein mutation capable of inhibiting the reaction downstream of the thioester intermediate and intercepting it with a synthetic peptide containing an N-terminal cysteine [83,84]. The synthetic peptide can in turn be a carrier for C-terminal site-specific protein modification. This has been successfully applied for fluorescein coupling and PEGylation [85,86]. EPL has also been used to generate monoclonal antibody conjugates [87,88]. Unfavorably for ADC generation reducing conditions have to be maintained to prevent oxidation of the accepting cysteine. For antibodies, Möhlmann et al. could maintain intact interchain disulfide bonds and retained binding properties [87], albeit with a product yield of 60%. PTS on the other hand is carried out by split inteins, first characterized in *Synechocystis* sp. PCC6803. This example consisted of separately expressed N- and C-inteins with high affinity towards each other fused to the N- and C-terminal half of the DnaE protein respectively. Once they assembled, self-cleavage similar to inteins took place forming a DnaE fusion protein [89]. This strategy also has been used in vitro and in the cellular environment with a protein transduction domain fused to the C-intein to enter the cell for labeling of the intracellular protein equipped with the N-intein [90]. The comparatively small size of the C-intein enables coupling of synthetic peptides similar to EPL. Product yields of up to 80% could be achieved using PTS with antibodies. Discovery and engineering of shorter C-intein (6 aa) and N-intein (12 aa) sequences has extended the applicability of the method to N-terminal protein modification [91,92]. Faster acting split inteins and inteins less sequence dependent on the extein sequence have since emerged [93,94]. For comfortable labeling with thiol reactive probes the Cys-Tag (EAGSCS) has been employed with split inteins [95]. Recently Bachman et al. modified nanobodies N-terminally with the help of split inteins and the Cys-Tag [96]. Han et al. generated bispecific IgG antibodies by producing full dimeric antibodies with a C-Intein instead of a second Fab-fragment. The Fab-fragment of second specificity was engineered with an N-intein and fused to the first antibody via PTS [97]. Downsides of both EPL and PTS are the long terminal inteins of approximately 100–150 aa that have to be appended to the protein of interest. While this might not be an issue for whole antibodies, or has been shown to be manageable for V_H_H, for some antibody mimetics this could lead to difficulties in production.

### 2.5. Tubulin Tyrosine Ligase

**Figure 7:**
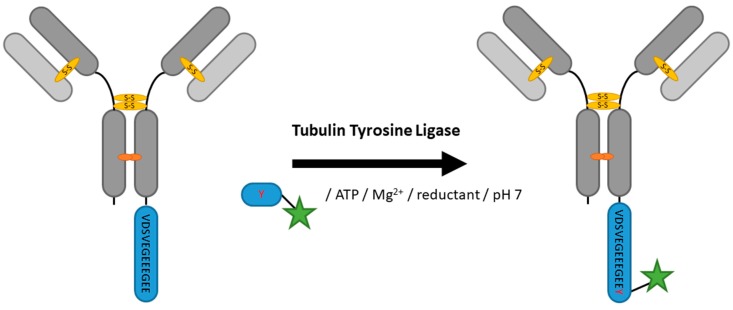
Scheme of an antibody modification by tubulin tyrosine ligase.

Catalyzed by tubulin tyrosine ligase (TTL), α-tubulin is modified post-translationally by C-terminal attachment of a tyrosine [98]. Banerjee et al. showed that TTL accepts tyrosine derivatives like formyl-tyrosine as a substrate [99]. For the use in other proteins, Schumacher et al. labeled a 13 aa peptide (VDSVEGEEEGEE), mimicking the C-terminus of α-tubulin, with TTL (Figure 7). This so-called Tub-Tag was introduced into proteins for efficient C-terminal modification of different proteins including an anti-GFP nanobody [100], achieving almost full conversion after 5 h of incubation. Substrate flexibility of TTL was further illustrated with, among others, unnatural and biotin-containing amino acids broadening the labeling chemistry spectrum [101]. Although limited to modification of the C-terminus and not yet used in context of full antibodies, TTL bears the potential for ADC generation. The tub tag’s predominant polarity could proof advantageous keeping in mind the solubility problems with mainly hydrophobic toxins during ADC generation.

### 2.6. Proteases (Trypsiligase and Subtiligase)

**Figure 8:**
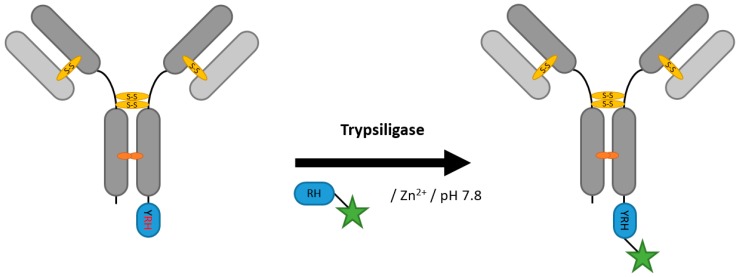
Scheme of an antibody modification by trypsiligase.

Proteases have been utilized for protein semi-synthesis for a long time and recently also for protein labeling. subtiligase, a variant of the serine protease subtilisin, though currently not used for ADC generation, is able to catalyze peptide formation, in addition to its hydrolytic activity [102]. Subtiligase has been improved by phage display for ligase activity [103]. Nonetheless, the problem of increased hydrolysis compared to ligase activity persists. Liebscher et al. engineered trypsiligase, a trypsin variant capable of terminal modification of proteins by cutting the short recognition sequence YRH and ligating proteins or peptides with an N-terminal RH moiety under the requirement of Zn^2+^ (Figure 8) [104]. C-terminal modification with PEG and fluorescein could also be achieved for an anti-Her2 Fab fragment, as well as coupling of the DM1 toxin with a yield of approximately 70% via a successive click reaction [105,106]. Though trypsiligase is only applicable to the C-terminus of the antibody, the only 3 aa long tag presents an advantage over most other mentioned enzyme strategies.

### 2.7. Phosphopantetheinyl Transferase

**Figure 9:**
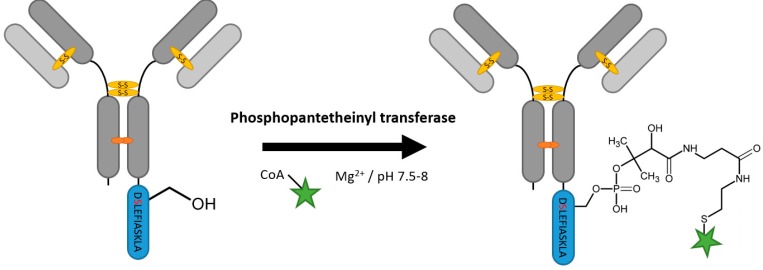
Scheme of an antibody modification by phosphopantetheinyl transferase.

Phosphopantetheinyl transferases transfer the phosphopantetheinyl moiety of coenzyme A to a specific serine residue in the target protein (Figure 9) [107]. Specifically, the Sfp Phosphopantetheinyl transferase (Sfp) from *Bacillus subtilis* is valued for protein labeling. Several peptide motives recognized by Sfp were found, among others the ybbR-tag (DSLEFIASKLA). The enzyme is able to transfer small molecule CoA conjugates to the tag serine positioned at the N- and C-terminus and also in flexible loops of the target protein [108,109,110]. Grünewald et al. recently generated antibody–drug conjugates site-specifically labeled at different loops in the constant region of trastuzumab with the help of different Sfp tags and achieved at least 95% conversion [111].

### 2.8. SpyLigase

**Figure 10:**
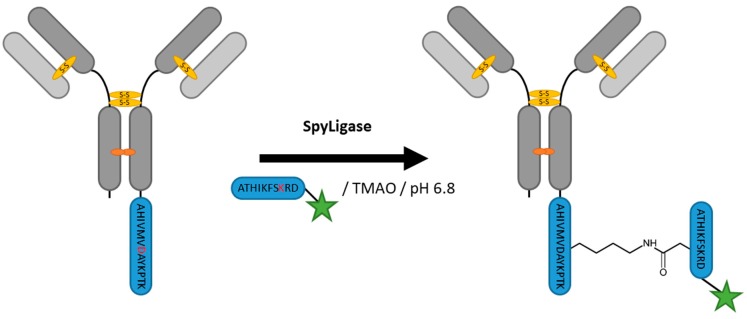
Scheme of an antibody modification by SpyLigase. TMAO, Trimethylamine N-oxide.

Zakeri et al. engineered the so-called SpyTag from the CnaB2 domain of fibronectin-binding protein from *Streptococcus pyogenes* [112]. The engineered SpyCatcher is able to mediate isopeptide formation of a side chain lysine and asparagine (Figure 10). This protein–peptide ligation method was refined by Fierer et al. by splitting the enzyme into three parts, SpyTag (AHIVMVDAYKPTK), K-Tag, (ATHIKFSKRD) and SpyLigase, which is able to fuse the two tags. They used this strategy to polymerize affibodies [113], and Siegemund et al. applied it for the generation of ADCs [114]. Both SpyTag and K-Tag are small, so this strategy can easily be applied to both protein termini. Furthermore, the low reaction temperatures, while still reaching 80% conversion with whole antibody substrates, are desirable for ADC production. The excess enzyme needed for the conversion remains a target for optimization.

### 2.9. Other Strategies

There are several further enzyme strategies that were adapted to antibody–drug conjugation or could be useful in this context based on their validated ability to ligate proteins. Lee et al. conjugated an anti-EGFR repebody by prenylation of a cysteine in a CaaX-motif with geranyl ketone pyrophosphate and successive oxime ligation with aminooxylated MMAF, reaching almost full conversion [115]. Lipoic acid ligase tagging of proteins and site-specific labeling with biotin ligase could be useful for antibody-drug conjugation as well [116,117,118]. *Escherichia coli* biotin ligase BirA has been used for protein labeling by Chen et al. with yields of up to 50% [117]. Comparably small amounts of enzyme are needed, yet the 15 aa tag is longer than that used in similar strategies. The ligase can attach a biotin analog to a lysine in the tag and the ketone group can then be labeled specifically. Heller et al. introduced a phosphocholination reaction for protein modification by AnkX. This *Legionella pneumophila* enzyme recognizes the peptide sequence TITSSYYR and adds a phosphocholine moiety from tagged CDP-choline to the second serine [119]. Conversion rates of up to 70% could be reached with AnkX, though it has not been used with antibodies or antibody fragment and mimetics thus far. GlycoConnect is a labeling strategy directed at glycostructures of the antibody. Derivatization is achieved by firstly trimming *N*-glycans with endoglycosidase to expose an *N*-acetyl glucosamine and secondly attach a click conjugatable *N*-acetyl galactosamine with glycosyltransferase. Using GlycoConnect, van Geel et al. could effectively generate nearly fully converted antibody–drug conjugates [120]. Elimination of the antibody’s *N*-glycosylation might have an effect on individual antibodies regarding blood clearance or ADCC, but the impact is in general less important for ADCs. The long incubation times at 37 °C and 30 °C are, however, not ideal for ADC production.

## 3. Conclusions

The sheer number and continuous development of new labeling strategies for antibody–drug conjugates demonstrates the interest in and importance of the issue. Besides the enzyme-based strategies discussed here, many parallel conjugation methods are being pursued, like the incorporation of unnatural amino acids and the design of chemically specific reaction sites in the antibody or antibody mimetic. Due to the various enzymes available for protein conjugation, chances are good that a suitable approach for a given protein of interest can be found as described in Table 1. Different and often short tags may be incorporated without disrupting structure and function. Problematic properties of antibody constructs, like stability or solubility, can be considered by testing various methods, which differ in buffer composition, additional chemical steps, or even the option to generate the conjugation ready antibodies by co-expressing the enzyme. Most of the enzymes mentioned here have already been used to generate homogenous antibodies at the laboratory scale. Nonetheless, it should be mentioned that a perfect or one-size-fits-all strategy remains elusive. Possible drawbacks include the demand for excess enzyme, reversible and incomplete reactions, or exclusively terminal ligation. Some of these issues have already been addressed and the different strategies could also go hand in hand for sequential labeling or labeling at different sites, e.g., with a toxin and a fluorophore or PEG. On the therapeutic side, a combination of antibodies and antibody mimetics, due to their individual characteristics, is conceivable as well. While current clinically investigated ADCs are mainly constructed using engineered cysteines, future generations of antibody–drug conjugates will likely include chemo-enzymatic strategies.

## Figures and Tables

**Table 1:** Overview of enzyme-based labeling strategies including substrate motif, reaction conditions, chemical strategy, site of modification, conversion yields with antibody or antibody mimetic substrates, drawbacks, and enzyme to substrate ratio.

Strategy		Substrate	Reaction Buffer	Duration	Labeling Strategy	Locus	Yields	Applications	Drawbacks	Enzyme/Substrate (Molar)
Formylglycine-generating enzymes		CXPXR	25 mM TEAM pH 9, 50 mM NaCl, 1 mM β-ME³	16 h at 18 °C	aldehyde coupling chemistry (HIPS-Ligation, trapped-Knoevenagel ligation etc.)	most surface accessible sites	85–95% ^a^ [44] ~95% ^b^ [40]	whole antibodies [37,39,40,44]	-long downstream coupling reaction -in vitro conversion at alkaline pH and reductive environment	0.1
Sortase A		LPXTG (G_n_)	50 mM Tris/HCl pH 7.9, 150 mM NaCl, 10 mM CaCl_2_	4 h at 42 °C	labeled peptide (G_n_ or LPXTG)	N- and C-terminal	~80% [65]	whole antibody [65,66] Fab [62]	-side reactions with proteins or peptides with terminal glycines	~1–3
Transglutaminase		(1) LLQGA or (2) GECTYFQAYGCTE	(1) 10 mM Phosphate buffer pH 7, 150 mM NaCl or (2) 100 mM HEPES pH 7	(1) 16 h at 37 °C or (2) 3 h at 25 °C	labeled alkyl- or oligo-amine	most surface accessible sites	~80–90% [78,79,81]	whole antibody [78,79,81]	-crosslinking via side chain lysine -deamidation of glutamine	(1) ~0.15–0.5 or (2) 1
Inteins	EPL	C-terminal intein (~100–150 aa)	50 mM HEPES/NaOH pH 8, 500 mM NaCl, 50 mM MESNA	22 h	labeled peptide with N-terminal cysteine	C-terminal	~60% [87]	whole antibody [87,88], V_H_H [96]	-long fusion tags -premature extein cleavage during expression reduces yields	N/A ^c^
	PTS	terminal intein (~100–150 aa)	50 mM HEPES/NaOH pH 7.5, 500 mM NaCl, 5 mM DTT	24 h	labeled short complementary intein (6–12 aa)	N- and C-terminal	~75% [87]	whole antibody [87,97]	-long fusion tags -hydrolysis or thiolysis during splicing yields side products	N/A ^c^
Tubulin Tyrosine Ligase		VDSVEGEEEGEE	20 mM MES/K pH 7, 100 mM KCl, 10 mM MgCl_2_, 2.5 mM ATP, 5 mM DTT	5 h at 37 °C	labeled tyrosine	C-terminal	99% [100]	V_H_H [100]	-limited to C-terminus -long tag	0.2
Trypsiligase		YRH	100 mM HEPES/NaOH pH 7.8, 0.1 mM ZnCl_2_, 100 mM NaCl, 10 mM CaCl_2_	1 h at 20 °C	labeled RH-peptide	C-terminal	70% [106]	Fab [105,106]	-remaining proteolytic activity generates side products	0.1
Phosphopantetheinyl transferase		DSLEFIASKLA	50 mM HEPES pH 7.5–8, 10 mM MgCl_2_	16 h at 20 °C	labeled Coenzyme A	N-, C-terminal and flexible loops	95% [111]	whole antibody [111]	-large linker -rather hydrophobic tag	0.4
SpyLigase		AHIVMVDAYKPTK	40 mM Na_2_HPO_4_, 20 mM Citric acid pH 6.8, 1.5 M Trimethylamine *N*-oxide (TMAO)	24 h at 4 °C	iso-peptide bond formation with labeled ATHIKFSKRD peptide	C- or N-terminal	~80% [114]	whole antibody [114], affibody [113]	-enzyme excess needed	~3
Farnesyltransferase		CVIM	50 mM Tris/HCl pH 7.4, 5 mM MgCl_2_, 10 µM ZnCl_2_, 5 mM DTT	12 h at 30 °C	attachment of aldehyde or keto functionalized prenyl pyrophosphate, subsequent oxime ligation	C-terminal	~95% [115]	repebody [115]	-long downstream coupling reaction at low pH -enzyme excess needed	~2
AnkX		TITSSYYR	20 mM HEPES pH 7.5, 50 mM NaCl, 1 mM MgCl_2_, 1 mM DTE	3 h at 25 °C	labeled cytidine diphosphate choline	N-, C-terminal and in internal loops	70% ^d^ [119]		-lower yields -reductive environment	0.02
Biotin ligase		GLNDIFEAQKIEWHE	50 mM Bicine pH 8.3, 5 mM Mg-acetate, 4 mM ATP	3 h at 30 °C	ligation of Biotin to side-chain of lysine and subsequent labeling of ketone group	N-, C-terminal and internal loops	~50% ^e^ [117]		-long Tag -lower yields	0.065–0.13
GlycoConnect		*N*-glycans	(1) 25 mM Tris pH 8, (2) 25 mM Tris/HCl pH 8, 10 mM MnCl_2_	(1) 16 h at 37 °C (2) 16 h at 30 °C	(1) trimming of glycan with endoglycosidase and (2) attachment of a conjugable GalNAc derivative by glycosyltransferase	*N*-glycans	>95% [120]	whole antibody [120]	-long incubation at >30 °C -disruption of *N*-glycans	(1) ~0.02 (2) ~0.015

^a^ In vitro conversion with bacterial formylglycine-generating enzymes (FGE). ^b^ Conversion of mAbs in human FGE co-expressing Chinese Hamster Ovary (CHO) cells. ^c^ Not a classical enzyme strategy since the inteins themselves bear endoprotease activity. Eukaryotic inteins are available, yet mostly prokaryotic inteins are used for expressed protein ligation (EPL) and protein trans-splicing (PTS). ^d^ Yield refers to reaction with tagged SUMO protein, not yet used in antibody or antibody mimetic context. ^e^ Yield refers to reaction with cyan fluorescent protein, not yet used in antibody context.
